# Identification of QTL associated with pod number in soybean

**DOI:** 10.3389/fgene.2026.1850332

**Published:** 2026-06-04

**Authors:** Yajun Xiong, Huan Yu, Alu Mao, Sawaira Jadoon, Zhiyu Liu, Kanglin Liu, Zhiqing Zhang, Yijie Chen, Bernard Gyebi-Nimako, Lijuan Qiu, Jun Wang

**Affiliations:** 1 The Shennong Laboratory, Zhengzhou, Henan, China; 2 Yangtze University, Jingzhou, Hubei, China; 3 State Key Laboratory of Crop Gene Resources and Breeding, Institute of Crop Sciences, Chinese Academy of Agricultural Sciences, Beijing, China; 4 Institute of Crop Molecular Breeding, Henan Academy of Agricultural Sciences, Zhengzhou, Henan, China

**Keywords:** candidate genes, genetic analysis, GWAS, pod number, soybean

## Abstract

Pod number is an important factor influencing soybean yield. In this study, a recombinant inbred line population derived from the cross between improved cultivar (*Glycine max*) and wild soybean (*Glycine soja*) was subjected to multi-environment phenotypic evaluation for pod number. Quantitative trait locus (QTL) mapping associated with pod number was performed utilizing both linkage analysis (LA) and genome-wide association study (GWAS) by integrating the phenotypic and genotypic data. The results showed that a total of 16 QTLs associated with pod number, notably, overlapping genomic intervals were observed between *qPN10-1* (by linkage analysis) and *qPN10-3* (by EMMAX), as well as between *qPN11-2* (by LA) and *qPN11-3* (by EMMAX), and these intervals were regarded as major-effect Quantitative trait locus regions. Furthermore, two candidate genes (*Glyma.10G206300*, and *Glyma.10G207500*) encode a basic Helix-Loop-Helix (bHLH*)* transcription factor and an armadillo (*ARM)*-repeat protein respectively, as determined through linkage disequilibrium analysis, SNP variant analysis, haplotype analysis, and gene functional annotation. These two candidates are possibly involved in the regulation of flower number, flowering time, and pod number (siliques number in *Arabidopsis thaliana*). These findings pave the way for gene cloning related to pod number, and provide novel insights into the genetic architecture underlying pod number variation in soybean.

## 1 Introduction

Yield in soybean is a comprehensive trait consisting of 100-seed weight, pod number, and seed number per plant. A strong correlation between pod number and yield has been previously established (r = 0.63, P < 0.01) (Berhanu et al., 2021). Numerous traits have been reported to contribute to pod number in soybean, such as plant height, branch number and flowering time. Research has shown that increased plant height correlates with a higher pod number in soybean (Liu et al., 2011). A significant positive correlation exists between branch number and pod number (P < 0.001) (Xu et al., 2021). Furthermore, flowering time is important for soybean pod number, as late-developing flowers are prone to abortion due to substantial assimilate consumption by early-developing pods (Egli and Bruening, 2000). The flower shedding rate also constitutes a key determinant of pod set (Raza et al., 2019). During the terminal flowering phase, soybean plants not only produce limited flowers but also exhibit flower shedding rates as high as 77.96% (Zhao et al., 2013). The physiological determination of pod number in soybean is influenced by numerous factors, including light exposure, defoliation, abscission, and phytohormone distribution. For instance, shading from the beginning of flowering (R1) to full maturity (R8) stages can reduce pod number (Jiang and Egli, 1993). Conversely, prolonged light exposure can significantly increase pod number; however, a greater impact has been observed at the late vegetative (V5) stage than at the early pod formation (R3) (Mathew et al., 2000). Defoliation of the lower part of soybean plant, usually caused by dense planting, also decreases pod number due to reduced light interception and diminished assimilation capacity (Board and Tan, 1995). Additionally, cytokinin can increase pod number per plant in soybean, presumably by altering assimilate partitioning and enhancing the “sink strength” of pods to prevent their abscission, thereby boosting yield (Nagel et al., 2001).

Due to its significant role in soybean yield, the genetic basis of pod number has been extensively studied. Using raw mean values across four environments, a total of 10 QTLs for the number of four-seeded pods at lower nodes in soybean were detected, accounting for 0.10%–2.94% of the phenotypic variance explained (PVE) (Raja et al., 2026). In addition, utilizing a RIL population, researchers mapped two quantitative trait loci (QTLs) (*qPN11-1 qPN6-11*) for pod number in soybean, which are located on chromosomes 11 and 12, respectively (Shim et al., 2017). A total of 602 QTLs associated with pod number were identified by a combination of linkage analysis (LA) and genome-wide association study (GWAS), and 11 candidate genes potentially involved in pod development and growth were identified (Song et al., 2020). Across six different environments, six QTLs associated with soybean pod number were identified in a recombinant inbred line (RIL) population (Charleston × Dongnong 594). Among those QTLs, *qpn-Chr34* accounted for 19.72% of the phenotypic variance explained (PVE) (Zhang et al., 2015). Using an RIL population (Qihuang 34 × Dongsheng 16) and a high-density SLAF (Specific-Locus Amplified Fragment)-based genetic map, two QTLs associated with pod number were identified across two environments. In 2024, a major-effect QTL was detected on chromosome 19, exhibiting an LOD score of 18.16 and explaining 23.02% of the PVE. Furthermore, 17 candidate genes were identified within this QTL region (Zhang et al., 2025). The genetic regulation of pod number in soybean involves genes expressed at specific developmental stages. Indeed, studies on pod number QTLs across different stages have demonstrated that some of these QTLs exhibit stage-specific expression patterns (Zhang et al., 2010; Sun et al., 2006). Several genes influencing pod number have been identified in soybean. For instance, the allelic variant *GmCYP78A10b* exerts an indirect negative regulation on pod number in soybean by conferring a positive effect on seed size/weight traits (Wang et al., 2015). The *GmAP1* gene potentially boosts pod yield in soybean through node number proliferation, suggesting a pathway for improving reproductive efficiency (Chen et al., 2020). *GmFULa* significantly increased soybean pod number (P < 0.01) by enhancing carbon assimilation to promote vegetative growth and biomass accumulation (Yue et al., 2021). Additionally, knocking out *GmST05* significantly reduced the number of pods per plant (P < 0.01) (Duan et al., 2022), while knockout of *GmEID1* resulted in increased pod number (Qin et al., 2023).

Although previous studies have identified multiple QTLs and genes associated with soybean pod number, its genetic architecture remains poorly understood—maybe due to the narrow genetic basis of cultivated soybean parents used in previous studies. In this study, we constructed a RIL population from a cross between the high-yielding cultivated soybean cultivar Zhongdou 41 and the wild soybean germplasm ZYD02878 to perform QTL mapping and predict candidate genes for pod number. This approach facilitates the discovery of favorable alleles and broadens the genetic diversity for breeding. These findings will provide an important theoretical foundation for subsequent cloning and functional validation of pod number-related regulatory genes and hold significant practical value for enhancing the efficiency of soybean yield genetic improvement.

## 2 Materials and methods

### 2.1 Plant materials and phenotyping

This study used the cultivated soybean Zhongdou 41 as the female parent and wild soybean ZYD02878 as the male parent to construct an advanced RIL population F_7,_ F_9,_ and F_11_, with a population size of 365 lines. Zhongdou 41, a main summer soybean cultivar in Hubei Province, was provided by the Oil Crops Research Institute of the Chinese Academy of Agricultural Sciences. ZYD02878, a wild soybean accession originating from Ningwu County, Shanxi Province, was obtained from the National Soybean Germplasm Bank. The parental lines exhibited significant differences in traits including seed coat color, number of pods per plant, resistance traits, and climbing growth habit. The population was planted in the summer of 2019 at the Yangtze University Science and Technology Industrial Park in Jingzhou City, Hubei Province (F_7_, 112°15′E, 30°36′N), in the summer of 2020 at the same location (F_9_), and in the summer of 2021 in Yucheng County, Shangqiu City, Henan Province (F_11_, 115°86′E, 34 41′N). These environments were labeled as 19JZ, 20JZ and 21SQ, respectively. Each RIL line was planted randomly with only one biological replicate per site. To prevent intertwining of soybean plants, a row spacing of 100 cm was adopted, and bamboo stakes were erected beside each plant during the early vegetative growth stage to guide the plants to climb vertically along the stakes. Individual plants were harvested separately, which facilitated subsequent trait measurement and prevented sample confusion. After the soybeans reached maturity, three plants were randomly selected from each line, and the number of effective pods was investigated in accordance with the Descriptors and Data Standard for Soybean (*Glycine* spp.) (Qiu and Chang, 2006).

Based on the 3σ principle and utilizing R packages including but not limited to outliers, car, nortest, fBasics, and Hmisc, outliers were eliminated. Subsequently, arithmetic means, Best Linear Unbiased Estimate (BLUE), and Best Linear Unbiased Predictions (BLUP) for each group of lines within individual environments were calculated using the R packages lmerTest, lme4, reshape2, and lsmeans. Descriptive statistics, correlation analysis, and normality tests were then performed with the lme4 and outliers packages in R.

### 2.2 Genotyping

Genomic DNA was extracted from young leaves collected at the V5 stage using the CTAB method (Doyle, 1990). Genotyping was conducted using the ZDX1 chip, which was designed based on resequencing data from 2,214 representative soybean accessions on the Illumina platform, with reference to the Wm82.a2.v1 genome and encompassing 158,959 SNPs (Sun et al., 2022). A total of 158,920 SNPs were obtained and after filtration out SNPs with integrity less than 0.8 and minor allele frequency (MAF) less than 0.05 using PLINK (Purcell et al., 2007), 74,805 SNPs were retained.

### 2.3 QTL analysis

QTL mapping was conducted in three environments (19JZ, 20JZ, 21SQ). Based on the mixed linear model (MLM), QTL mapping analysis was performed separately using Efficient Mixed-Model Association eXpedited (EMMAX) (Kang et al., 2010) and IciMapping 4.2 software. The kinship matrix for EMMAX analysis was calculated using the emmax-kin-inter64 program. The significance thresholds were set to -lg (1/total SNPs). The QTL interval was defined as the region extending 100 kb both upstream and downstream of the significant locus. Manhattan and Q-Q plots were generated using the CMplot package in R v 4.3.2. In IciMapping 4.2, the map function was used to construct the genetic map. QTL mapping was performed using the inclusive composite interval mapping (ICIM) method, with a mapping step size of 1.00 cM, a PIN of 0.001, and Permutation tests were employed to empirically determine the LOD threshold, with 1,000 iterations performed for the pod number per plant phenotype in each planting environment at a significance level of P = 0.05. Consequently, the genome-wide significance LOD threshold was established as 2.5.

### 2.4 Candidate gene identification

QTL regions mapped across multiple environments (19JZ, 20JZ, and 21SQ) were selected for candidate gene identification. Candidate gene analysis was performed using four different approaches, including linkage disequilibrium (LD) analysis (Shin et al., 2006), SNP variation, haplotype, and gene functional annotation analyses were conducted. First, Using the Wm82.a2.v1 genome as the reference, significant loci were selected, and the flanking 100 kb regions on each side were defined as candidate intervals for LD analysis. The target intervals were refined based on an *r* ^2^ threshold (>0.8). Subsequently, SNP variant analysis was performed to further screen and retain SNPs resulting in nonsynonymous mutations, premature termination (stop-gain), readthrough (stop-loss), and alternative splicing. Haplotype analysis was then performed on genes within the narrowed candidate intervals based on the genotypic and phenotypic data of a NAM population consisting of 2,443 RILs (Song et al., 2024) to examine the significance of phenotypic differences among haplotypes. Phenotypic differences between distinct haplotypes were assessed using the Wilcoxon rank-sum test. Haplotype analysis was conducted using the HaploAssistant package (Yu et al., 2023), and haplotypes with frequencies below 30 were excluded from subsequent analysis due to insufficient statistical power. Finally, gene functional annotation was performed on the candidate genes using data from the SoyBase database (https://www.soybase.org/) and the Phytozome database (https://phytozome.jgi.doe.gov/; assembly Wm82.a2.v1) to identify genes regulating the number of pods per plant in soybean.

## 3 Results

### 3.1 Phenotypic analysis of pod number

**FIGURE 1:**
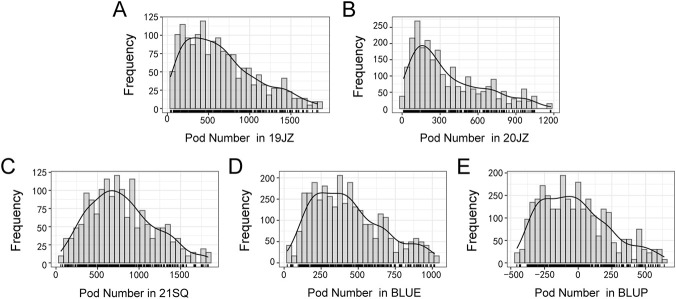
Phenotypic distribution of the pod number in different environment. **(A)** 19JZ. **(B)** 20JZ. **(C)** 21SQ. **(D)** The Best Linear Unbiased Estimate (BLUE). **(E)** the Best Linear Unbiased Prediction (BLUP).

Among the phenotypic values (mean, BLUE, and BLUP) obtained from the three environments (19JZ, 20JZ, and 21SQ), the wild parent ZYD02878 (P2) exhibited a consistently higher pod number than the cultivated parent Zhongdou 41 (P1) across all environments, with the most pronounced difference observed in the 21SQ environment (1934.00 for P2 vs. 128.00 for P1). The largest range of pod number variation was observed in the 19JZ environment (37.00–1,827.00), indicating the greatest phenotypic divergence among lines, whereas the smallest range occurred in the 20JZ environment (11.00–1,197.33). The highest mean pod number per plant was recorded in the 21SQ environment, while the 20JZ environment exhibited greater within-population variability. Transgressive segregation was observed for all measured values Skewness values were all positive (ranging from 0.47 to 0.92), indicating right-skewed distributions across all environments, i.e., most lines produced fewer pods, while a small number of lines exhibited extremely high pod numbers. Kurtosis values were all negative (ranging from – 0.07 to – 0.38), suggesting relatively dispersed data distributions. (Table 1). The frequency distribution of pod number in the 21SQ environment was approximately normal, whereas the distributions for the 19JZ and 20JZ environments, as well as the BLUE and BLUP values, were right-skewed (Figure 1). Correlation analysis revealed that pod number had significant, albeit moderate, positive correlations among the three environments (r = 0.49–0.59; P < 0.01) (Table 2).

**TABLE 1:** Phenotypic statistics of pod number.

Environments	Parent		Population							
	P1	P2	Min	Max	Mean	SD	CV	Skew	Kurtosis	P value
19JZ	107.00	1727.00	37.00	1827.00	647.78	423.18	0.65	0.73	−0.24	<0.001
20JZ	49.00	1748.00	11.00	1197.33	362.39	280.00	0.77	0.92	−0.07	<0.001
21SQ	128.00	1934.00	58.33	1833.00	793.66	389.53	0.49	0.47	−0.36	<0.001
BLUE	118.62	1048.09	83.05	1578.05	647.20	337.21	0.52	0.61	−0.38	<0.001
BLUP	−429.93	196.71	−455.02	638.46	−24.04	250.04	—	0.56	−0.37	<0.001

**TABLE 2:** Correlation analysis of pod number in different environments.

Environment	19JZ	20JZ	21SQ
19JZ	1	​	​
20JZ	0.57**	1	​
21SQ	0.49**	0.59**	1

**Represents significance level of 0.01.

### 3.2 QTL mapping of pod number

**FIGURE 2:**
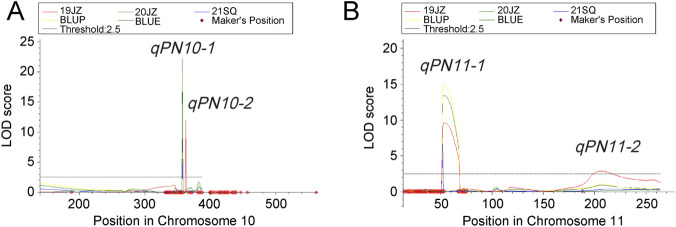
Linkage analysis of pod number (ICIM). **(A)** Chromosome 10. **(B)** Chromosome 11.

A total of 11 QTLs associated with pod number were mapped to chromosomes 3, 5, 6, 10, 11, 12, 14, and 15 using ICIM (Supplementary Figure S1). The LOD scores of these QTLs ranged from 2.68 to 16.91, and the phenotypic variance explained (PVE) ranged from 1.79% to 21.99%. Among them, five QTLs (*qPN5-1*, *qPN6-1*, *qPN10-1*, *qPN11-2,* and *qPN12-1*) were consistently detected across multiple environments, while the remaining QTLs were detected only in single environment (Table 3). Specifically, *qPN5-1*, *qPN10-1* and *qPN11-2* were detected as major-effect QTL in multiple environments with phenotypic variance explained exceeding 10% (Figure 2).

**TABLE 3:** QTL mapping of pod number by ICIM.

QTL name	Chromosome	Start	End	ADD	PVE/%	LOD value	Environment
*qPN3-1*	3	36055334	36093981	−35.37	1.97	2.69	BLUP
*qPN3-2*	3	36717177	36793579	−65.80	2.79	2.91	21SQ
*qPN5-1*	5	37167537	37481276	70.92	18.16	3.39	20JZ, BLUE, BLUP
*qPN6-1*	6	3709181	4138913	51.43	2.32	2.82	21SQ, BLUE, BLUP
*qPN10-1*	10	45243194	45322107	129.70	21.99	16.91	20JZ,21SQ, BLUE
*qPN10-2*	10	46156099	46350300	119.69	5.48	5.01	19JZ
*qPN11-1*	11	31891786	31901505	−148.50	8.49	5.18	19JZ
*qPN11-2*	11	10904384	11099081	−91.30	13.36	14.83	21SQ,19JZ, BLUE, BLUP
*qPN12-1*	12	5475077	5817926	6.75	7.03	9.11	21SQ, BLUP
*qPN14-1*	14	5929565	6943720	−71.89	3.39	2.68	21SQ
*qPN15-1*	15	10851120	10873304	117.14	7.91	2.75	21SQ

**FIGURE 3:**
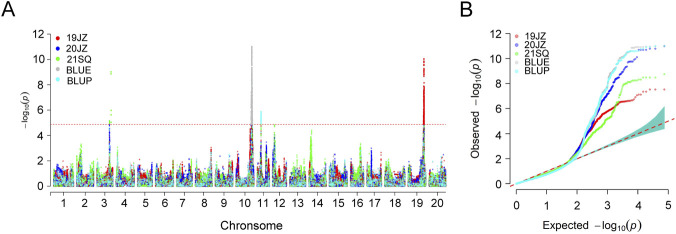
Integrated Manhattan and QQ plot of EMMAX analysis of the pod number. **(A)** Manhattan plot. **(B)** QQ plot.

EMMAX-GWAS identified five QTLs, four of which were repeatedly detected across multiple environments, with the exception of *qPN3-4*, which was observed only in a single environment (20JZ) (Table 4) (Figure 3). In addition, three QTLs (*qPN3-4*, *qPN10-3*, and *qPN11-3*) overlapped with *qPN3-1*, *qPN10-1*, *qPN10-2*, and *qPN11-2* mapped by ICIM, those QTLs were co-localized.

**TABLE 4:** QTL mapping of pod number by EMMAX.

QTL name	Chromosome	Start/	End	Number of genes	Environment
*qPN3-3*	3	40420694	40771281	50	19JZ,20JZ,21SQ, BLUE, BLUP
*qPN3-4*	3	35914456	36409036	49	20JZ
*qPN10-3*	10	43350192	46990641	391	19JZ,20JZ,21SQ, BLUE, BLUP
*qPN11-3*	11	10715099	11238789	54	19JZ,20JZ,21SQ, BLUE, BLUP
*qPN19-1*	19	43837861	46274815	245	19JZ,21SQ, BLUE, BLUP

### 3.3 Candidate gene analysis

**FIGURE 4:**
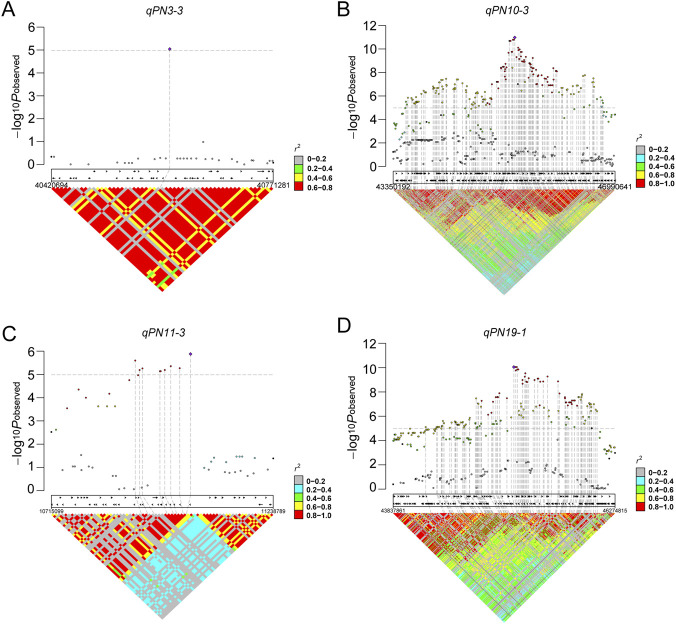
Linkage disequilibrium analysis of important QTL region. **(A)** *qPN3-3*. **(B)** *qPN10-3*. **(C)** *qPN11-3*. **(D)** *qPN19-1*.

Among the 16 QTLs associated with pod number, four unique QTL regions (*qPN3-3*, *qPN10-3*, *qPN11-3*, and *qPN19-1*) that were consistently mapped across three environments (19JZ, 20JZ, and 21SQ) were selected as candidate QTL for further analysis. A total of 777 genes were annotated within those four QTL regions. The *qPN3-3* region was excluded from subsequent analysis because it was defined by only a single SNP that exceeded the significance threshold and therefore failed to satisfy our filtering criteria. The sizes of the remaining three candidate regions (*qPN10-3*, *qPN11-3*, and *qPN19-1*) were 3,640,449 bp, 9,476,310 bp, and 2,101,180 bp, respectively. Following LD analysis, these intervals were refined to 1,697,290 bp, 72,147 bp, and 850,158 bp, which collectively contained 302 genes (Figure 4).

SNP variant analysis identified 86 genes harboring SNP variants, which corresponded to 127 mutation sites within the narrowed intervals. These included 16 nonsynonymous, 41 synonymous, 32 UTR, and 13 upstream/downstream SNPs, respectively (Supplementary Table S1).

In the NAM population, the number of SNPs within the coding sequence (CDS) regions of candidate genes across the three candidate intervals (qPN10-3, qPN11-3, and qPN19-1) ranged from 1 to 23, encompassing four variant types: nonsynonymous, synonymous, premature stop codon, and UTR mutations (Supplementary Table S2). After filtering low-frequency haplotypes, a maximum of five haplotypes per gene were identified. Hap1 was the predominant haplotype, represented in the highest number of accessions, accounting for 49.57%–95.66% of the total samples. This was followed by Hap2, which accounted for 1.23%–35.2% (Supplementary Table S3). Association analysis between gene haplotypes and pod number revealed significant phenotypic differences among haplotypes for 124 genes (Supplementary Table S4).

Functional annotation identified two key candidate genes potentially regulating pod number (Table 5). *Glyma.10G206300* is an ortholog of *AT1G31050*, encoding a basic helix-loop-helix (bHLH) DNA-binding protein with protein dimerization activity. *Glyma.10G207500* encodes an ARM repeat-containing protein involved in intracellular protein transport, showing orthologs to the *Arabidopsis* gene *AT2G35630*.

**TABLE 5:** Functional annotation of candidate genes.

Gene	Homologue	Annotation
*Glyma.10G206300*	*AT1G31050*	Basic helix-loop-helix (*bHLH*) DNA-binding superfamily protein
*Glyma.10G207500*	*AT2G35630*	*ARM* repeat superfamily protein

**FIGURE 5:**
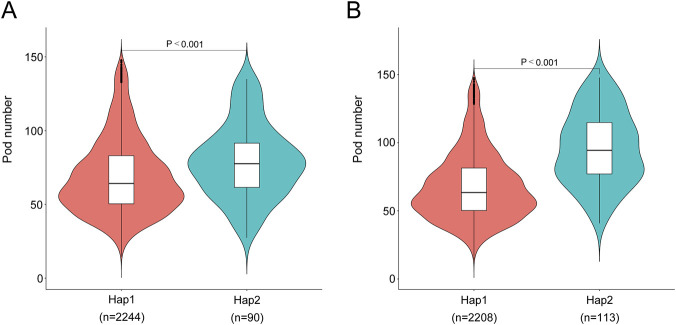
Phenotypic comparison of haplotypes of candidate gene. **(A)** *Glyma.10G206300*. **(B)** *Glyma.10G207500*.

The results of the haplotype-phenotype association analysis for the candidate genes showed that both candidate genes, *Glyma.10G206300* and *Glyma.10G207500*, harbored two haplotypes. Highly significant associations (Wilcoxon test, P < 0.01) were observed between both Hap1 and Hap2 within each candidate gene and the phenotype (Figures 5A,B).

## 4 Discussion

In this study, a RIL population derived from a cross between the wild soybean ZYD02878 and the cultivated soybean Zhongdou 41 was subjected to multi-environment phenotypic evaluation. By integrating LA and GWAS, candidate genes were identified, and their potential roles in flower and fruit development were validated through functional analysis of their homologous genes in *Arabidopsis thaliana*. This study broadens the genetic basis of soybean, overcoming the limitations imposed by the narrow genetic background of cultivated soybean parents used in previous studies. The findings provide a theoretical foundation and practical tools for marker-assisted selection, gene editing, and high-yield soybean breeding targeting pod number, thereby holding significant theoretical and practical value for improving soybean yield and ensuring food security.

Wild soybean and cultivated soybean are closely related without reproductive isolation. Numerous studies have utilized wild soybeans for QTL mapping and gene mining related to agronomic traits (Chen et al., 2023; Li et al., 2022; Wang et al., 2024; Yang et al., 2019; Yang et al., 2025). Previous research has shown transgressive segregation for quality and yield traits in hybrid offspring populations of wild and cultivated soybeans (Wang et al., 2024; Yang et al., 2025), which is consistent with the findings in this study.

In this study, 16 QTLs associated with pod number were identified. Among these, *qPN10-1* colocalizes with *qPNLD-10-3,* which is responsible for four-seed pods in the lower part of soybean plants. This QTL was identified using a cultivated soybean-derived RIL population for pod number-related QTL analysis (Song et al., 2020). Additionally, *qPN19-1* coincides with a QTL (*qBR19-1)* associated with soybean branching (Shim et al., 2017).

This study identified two candidate genes potentially regulating pod number in soybean. *Glyma.10G206300* encodes a basic helix-loop-helix (bHLH) protein. In *Arabidopsis*, bHLH transcription factors play significant roles in seed germination, flowering time, and abiotic stress response. For example, the *Arabidopsis* gene ortholog *AT1G31050*, which encodes a bHLH transcription factor, has been shown to influence multiple developmental processes: knockout of this gene promotes primary root growth, increases flower number, shortens internode length, and delays flowering (Makkena and Lamb, 2013) *GmbHLH98* is upregulated under drought and salt stress, and its overexpression enhances seed protein content in transgenic plants, demonstrating the potential of bHLH transcription factors in improving both yield and quality in soybean (Wu et al., 2025). Another *Arabidopsis* homologue of *Glyma.10G206300*, *SPATULA* (*SPT*), also supports its potential role in fruit development: loss-of-function *SPT* mutants produce significantly fewer siliques than wild-type plants (Groszmann et al., 2008). These functional insights suggest that *Glyma.10G206300* may be a key regulator of pod number in soybean. *Glyma.10G207500* encodes an *ARM* repeat protein. In soybean, *GmDFB1—*another *ARM* repeat protein—was reported to interact with *GmSK17* and participate in siRNA suppression by blocking *GmAGO1*-mediated mRNA cleavage, ultimately leading to reduced miRNA accumulation. Phenotypically, the *gmdfb1* mutant exhibits severe floral structural defects that result in a complete absence of pod formation (Li et al., 2024). The *Arabidopsis* homologue of *Glyma.10G207500, MOR1* (*AT2G35630*), shares 85.7% sequence identity. Knockout mutants of *MOR1* exhibit dwarfism, failure to flower, and lethality at temperatures above 28 °C, although normal growth, flowering, and ploidy are restored when plants are returned to optimal temperatures (Whittington et al., 2001). Additionally, mor1 mutants display temperature-sensitive anther indehiscence and impaired flowering (Liu and Tan, 2022). Therefore, both candidates, *Glyma.10G207500* and *Glyma.10G206300,* were likely to play significant roles in pod number formation, warranted further investigation.

In this study, a recombinant inbred line (RIL) population derived from the cross between the cultivated soybean (Zhongdou 41) and wild soybean (ZYD02878) was constructed. Combined with multi-environment phenotypic evaluation and multiple QTL mapping approaches, a total of 16 QTLs associated with pod number per plant in soybean were identified. Subsequently, by employing LD analysis, SNP variant analysis, haplotype analysis, and gene functional annotation, two candidate genes, *Glyma.10G206300* and *Glyma.10G207500*, were screened out as key regulators of soybean pod number. These two genes encode a bHLH transcription factor and an ARM repeat protein, respectively. This discovery not only provides new genetic resources and molecular markers for high-yield molecular breeding in soybean and broadens the genetic basis for breeding, but also lays a theoretical foundation for the subsequent cloning and functional validation of pod number-related genes. Furthermore, it holds significant theoretical and practical importance for improving the efficiency of genetic enhancement of soybean yield and ensuring the security of soybean production.

## 5 Conclusion

Utilizing a RIL population derived from Zhongdou 41 × ZYD02878, we performed QTL mapping and candidate gene prediction were performed based on mean pod number per plant across three environments, along with BLUE and BLUP values. In this study, 16 QTLs were identified, among which *qPN3-2*, *qPN10-1*, and *qPN11-1* were consistently detected by both methods. Notably *qPN10-1* and *qPN19-1* co-localized with previously reported loci and were detected across multiple environments. Additionally, two candidate genes, *Glyma.10G206300* and *Glyma.10G207500*, were predicted to indirectly affect soybean pod number by mediating flowering time and flower number. These findings establish a foundation for the cloning and functional characterization of pod number-associated genes in soybean.

## Data Availability

The original contributions presented in the study are publicly available. This data can be found in the Soybean Functional Genomics and Breeding repository at https://sfgb.rmbreeding.cn/about/data.

## Supplementary material

SUPPLEMENTARY FIGURE S1 Linkage analysis of pod number (ICIM).
