# Supplementary figures and images for: Identification of QTL associated with pod number in soybean
Source: Front Genet. 2026 Jun 4;17:1850332. doi: 10.3389/fgene.2026.1850332 (PMC13275047; doi:10.3389/fgene.2026.1850332)

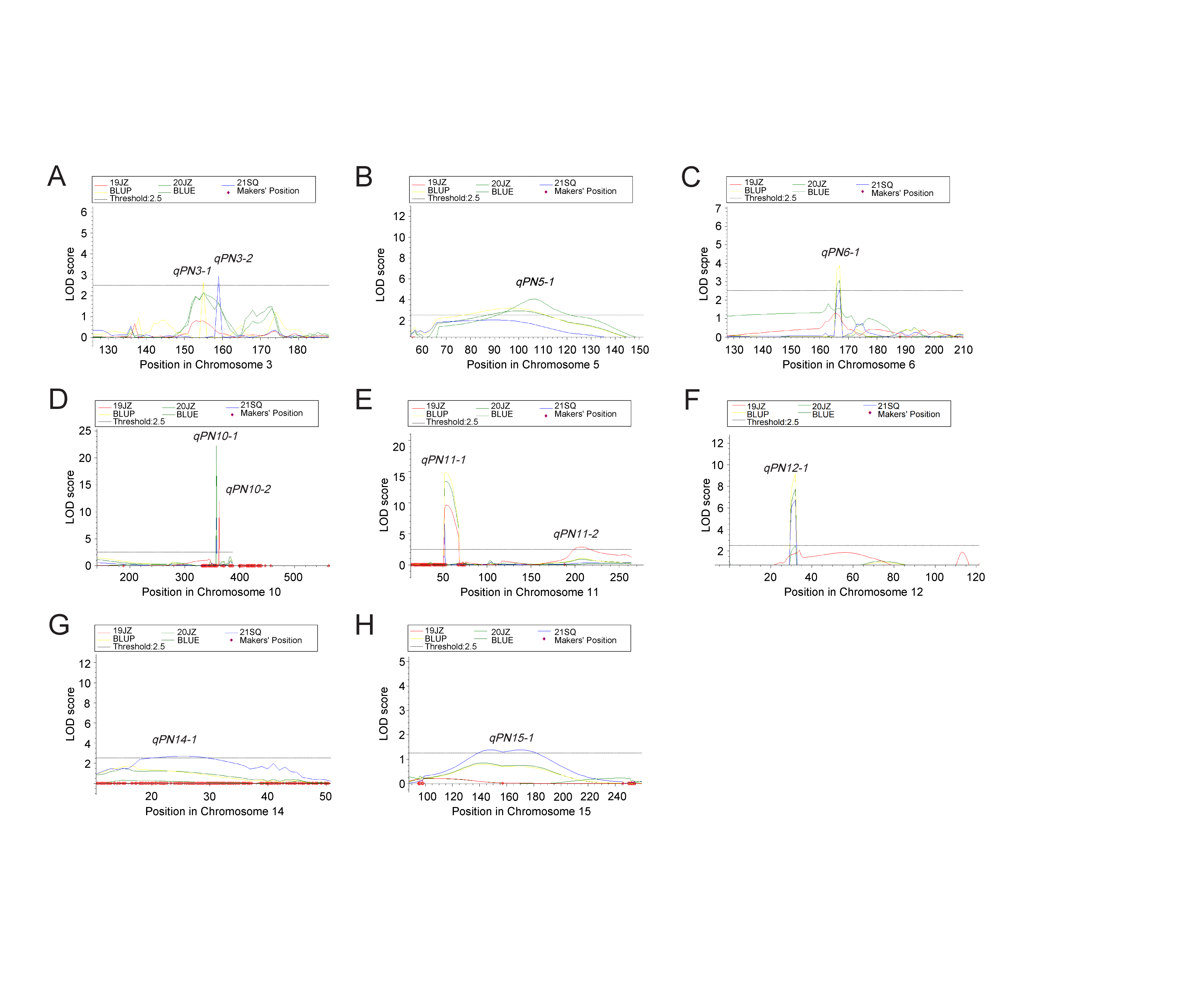

Supplement: Supplementary file 3 [file Image1.tif]
